# Temporal dynamics of inflammatory, platelet, and neurotrophic markers during social stress in relation to suicidal ideation and suicide attempt history

**DOI:** 10.1016/j.bbih.2025.100984

**Published:** 2025-03-24

**Authors:** Aiste Lengvenyte, Emilie Olié, Fabrice Cognasse, Hind Hamzeh-Cognassse, Adrian Alacreu-Crespo, Philippe Courtet

**Affiliations:** a Department of Emergency Psychiatry and Acute Care, Lapeyronie Hospital, CHU Montpellier, Montpellier, France; b Institute of Functional Genomics, University of Montpellier, CNRS, INSERM, Montpellier, France; c Université Jean Monnet, Mines Saint-Étienne, INSERM, U 1059, Sainbiose, Saint-Étienne, France; d Etablissement Français du Sang Auvergne-Rhône-Alpes, Saint-Étienne, France; e Department of Psychology and Sociology, University of Zaragoza, Zaragoza, Spain

**Keywords:** Inflammation, Suicide, Biomarkers, Stress response, Acute interpersonal stress, Platelet activation, Neurotrophic factors, Temporal dynamics, Trier social stress test

## Abstract

Interpersonal stress is a major precipitant of suicidal ideation (SI) and suicide attempts (SA), yet the underlying biological mechanisms remain unclear. This study examined dynamic plasma concentrations of inflammatory, platelet activation, and neurotrophic markers during acute social stress in 76 depressed women (mean age: 39.7 ± 1.39 years) with and without SA history or recent SI. Participants underwent the Trier Social Stress Test, with biomarkers measured at five time points before, during, and after the stressor. Covariate-adjusted growth curve models and area under the curve analyses were applied. SI was associated with elevated overall TNF-α levels (0.82 ± 0.05 vs. 0.61 ± 0.05, p = 0.039). Participants without SI showed cubic trajectories in TNF-α (t_228_ = 4.76, p < 0.001) and MIP-1β (t_235_ = 2.12, p = 0.035), and quadratic trajectories in platelet markers TSP-1 (t_274_ = −2.42, p = 0.016), PF-4 (t_274_ = −2.25, p = 0.025), and NAP-2 (t_274_ = −2.43, p = 0.016), which were absent in participants with SI. Cubic patters in inflammatory responses were also observed in participants without SA history, but not in suicide attempters (TNF-α: t_228_ = 3.15, p = 0.030; MIP-1β: t_235_ = 2.74, p = 0.007). Meanwhile, participants with SI or SA, but not those without, showed a linear BDNF increase (t_275_ = 2.39, p = 0.017; t_275_ = 2.28, p = 0.024, respectively). These findings suggest that SI and SA may be associated with impaired dynamic immune-inflammatory, platelet, and neurotrophic systems responses to acute interpersonal stress, reflecting systemic biological rigidity.

## Highlights

• Suicidal ideation was associated with elevated overall TNF-α levels.
• Suicide attempters and ideators showed less dynamic inflammatory stress responses.
• Platelet activation responses to stress were blunted in individuals with suicidal ideation.
• BDNF increased linearly in participants with suicidal ideation or suicide attempts.
• Findings suggest impaired biological flexibility under stress in suicidal individuals.

## 1 Introduction

Acute interpersonal stress is a well-established trigger for suicidal thoughts and behaviours (STB) in vulnerable individuals (Massing-Schaffer et al., 2019). However, although social stress is widespread, only a small subset of individuals, even within clinical populations, progresses to STB. This discrepancy can be understood within the stress-diathesis framework of suicide, which posits that STB results from an interaction between proximal stressors and biologically ingrained vulnerabilities (diathesis) (John Mann and Rizk, 2020).

Increasing evidence indicates that individuals with STB have altered levels of immune-inflammatory markers, with potentially reflects this underlying diathesis (Lengvenyte et al., 2024; Neupane et al., 2023). Recent findings also indicate disruptions in markers of vascular homeostasis and platelet function among individuals with STB (Lengvenyte et al., 2023). Neurotrophic factors, such as brain derived growth factor (BDNF), have also been proposed to be altered in STB (Fusar-Poli et al., 2021). However, much of this research has relied on cross-sectional designs, failing to capture the dynamic biological shifts in response to stress in at-risk individuals. Acute inflammatory responses to stress are inherently dynamic, involving complex cascades of upregulation, migration, and downregulation of molecular processes (Megha et al., 2021). While these fluctuations are known to be critical in physical injury recovery, they remain underexplored in psychological stress and STB research, potentially hindering deeper insights into STB pathophysiology.

Theoretical General Escape Theory of suicide and mathematical modelling of suicide as a complex dynamical system have shown that suicidal thoughts emerge when alternative escape behaviours failed to effectively regulate aversive internal states (Kleiman et al., 2018; Wang et al., 2023). It underscores the need to study STB-related biological phenomena during these experiences. Previous research has pointed to a dysregulation of the hypothalamic-pituitary-adrenal (HPA) axis as a potential contributor to STB, often presenting as a blunted cortisol response (Eisenlohr-Moul et al., 2018; O'Connor et al., 2017; Shalev et al., 2019). Acute interpersonal stress can alter glucocorticoid sensitivity in cytokine production, increase inflammation-related immune activity, and activate brain regions implicated in social rejection and negative affect processing (Slavich et al., 2010; Strahler et al., 2015). Increased inflammation during social stress tasks has been linked to heightened amygdala activity and stronger coupling between amygdala and dorsomedial prefrontal cortex coupling, potentially amplifying threat responses (Muscatell et al., 2015). Additionally, elevated translocator protein 18k Da (TSPO) binding, a marker of neuroinflammation, has been associated with vulnerability to stress-induced increases in suicidal ideation (SI) and negative affect in depression (Herzog et al., 2024).

The Social Signal Transduction Theory suggests that the immune system has evolved to detect social threats, such as rejection or isolation, as precursors to physical harm, thereby initiating preparatory inflammatory responses (Slavich, 2020). Laboratory-based social stress can induce increase in pro-inflammatory cytokines in both healthy volunteers and people with depression. While studies have showed heterogenous results, and there seems to be biomarker-specific differences, meta-analytical evidence has suggested that people with depression may have a blunted response to in a classic pro-inflammatory cytokine interleukin-6 (IL-6), compared to healthy controls, suggesting that clinical populations might have reduced biological reactivity to social stress (Annam et al., 2024; Marsland et al., 2017). Meanwhile it has been shown that baseline concentrations of pro-inflammatory cytokines are associated with cerebral activation during a social exclusion task (Conejero et al., 2019). Some studies have linked STB-related factors, including early life adversity, perceived lower social standing, and isolation, with elevated peripheral inflammatory cytokine responses during social-evaluative stress (Hackett et al., 2012; John-Henderson et al., 2015; Pace et al., 2006). Notably, a study in adolescent females found that the absence of saliva cytokine reactivity following interpersonal stress was associated with increased risk for STB over the next nine months (Clayton et al., 2023). However, studies on inflammatory responses to social stress in STB remain scarce, and there are no studies also considering neurotrophic and platelet markers.

This study examines the temporal trajectories of inflammatory cytokines, chemokines, neurotrophic factors, and markers of platelet activation and vascular function during a controlled social stressor in women with depression history with and without recent SI or a history of suicide attempt (SA), accounting for individual variations in stress reactivity. By focusing on real-time biomarker fluctuations during acute stress, this research moves beyond static measures to capture the dynamic biological processes that may underlie suicidal crises. We hypothesized that women with SA history and recent SI would have higher overall levels, but reduced variability of pro-inflammatory cytokines, platelet activation, and neurotrophic markers.

## 2 Material and methods

### 2.1 Study design and participants

This monocentric, cross-sectional, case-control study recruited seventy-six adult women (mean age ± SD = 39.7 ± 1.39 years) at the Department of Psychiatric Emergency and Acute Care at the Academic Hospital of Montpellier, France, a center specialized in mood disorders and suicidal crisis management. Participants were required to have a diagnosis of a major depressive episode according to the Diagnostic and Statistical Manual 5 (DSM-5) criteria, to be socially insure, and to be able to provide informed consent. Participants were divided into two groups: those with (N = 44) and without (N = 32) lifetime history of SA. SA was defined per the lifetime Columbia Suicide Severity Rating Scale (C-SSRS) as a self-injurious act with at least partial intent to die (Posner et al., 2011). Additionally, 36 participants reported recent suicidal ideation (SI), defined by positive answer to the Columbia Suicide Severity Rating Scale, suicidal ideation subscale second question “Have you actually had any thoughts of killing yourself?” in the past eight days, while 40 did not.

Exclusion criteria were: (1) any history of schizoaffective disorder or schizophrenia, (2) current hypomania or mania, (3) current eating disorder, (4) substance or alcohol use disorder within the last 12 months, (5) any medical condition or treatment known to affect the acute stress response (e.g. Cushing's syndrome or corticosteroids), (6) current use of anti-inflammatory drugs or antibiotics, (7) pregnancy or lactation, verified by urinary pregnancy test (participants were administered an urinary pregnancy test to ensure this).

The protocol was approved by the CPP Montpellier Sud-Méditerranée IV Ethics Committee at CHU Montpellier (approval number 15.073, date 24/09/2015) and adhered to the Declaration of Helsinki. All participants provided informed consent and received 50 euros for participating in two sessions (clinical assessment and stress test).

### 2.2 Clinical assessment

At inclusion, a trained psychiatrist conducted interviews to collect sociodemographic data, gynecological, medical, treatment history, and smoking status. Current psychiatric disorders were evaluated using DSM-5 criteria. SA history and recent SI were assessed with the C-SSRS, evaluating lifetime and past 8 days SI and SA (Posner et al., 2011). Depression severity was evaluated with the clinician-rated Inventory of Depressive Symptomatology (IDS-C) (Rush et al., 1996). Childhood trauma history was assessed with the brief Childhood Trauma Questionnaire (CTQ) (Bernstein et al., 2003). Height and weight measurements were recorded for body mass index (BMI) calculations. The second visit to perform the Trier Social Stress test was scheduled within 7 days after the initial evaluation. Participants were instructed to maintain their usual dietary and sleep habits and to avoid heavy physical exercise, alcohol, and stimulants (including coffee and tea) on the day prior to the stress task.

### 2.3 Trier Social Stress Test

All social stress sessions were scheduled between 1:00 p.m. and 5:00 p.m. to reduce the impact of circadian rhythms. Upon arrival, participants were placed in the experimental room, where they were explained the study task. A venous cannula with a saline lock was inserted into the antecubital fossa for blood sampling. An initial baseline sample was taken (−10 min), followed by a 10-min waiting period before the stressor task.

All participants then underwent the TSST (Kirschbaum et al., 1993), a validated psychosocial stressor. During the task, after a 3-min preparation, participants had to give a 5-min speech on their professional qualifications for a job of participant's choice, followed by a 5-min mental arithmetic task, both performed in front of a neutral, non-response panel (one male and one female), dressed in white blouses. The participants were informed the session would be video-recorded, with visible camera and microphone equipment, but recordings would later be discarded.

### 2.4 Blood collection and biochemical assays

Blood samples were collected into EDTA tubes from a venous cannula at one pre-stressor (−10 min) and four post-stressor time points (+30, +60, +90, +120 min) to capture dynamic changes in inflammatory, platelet activation, and neurotrophic markers. Samples were immediately centrifuged at 3000×*g* for 20 min at room temperature to separate plasma, which was then aliquoted and stored at −80 °C for batched analysis. Each analyte was measured in duplicate, using 50 μl of plasma for each assay. Standard curves were generated, and the lower limits of detection (LOD) were defined according to manufacturer specifications. Values below the LOD were considered undetectable.

Plasma concentrations of inflammatory, platelet activation, and neurotrophic markers were measured. Specifically, inflammatory markers included tumour necrosis factor-α (TNF-α), interleukin-6 (IL-6), macrophage inflammatory protein-1β (MIP-1β), and soluble interleukin-2 receptor α (sIL-2Rα). Due to undetectable baseline IL-6 levels in 53 % of patients (N = 40), IL-6 was excluded from further analyses. Platelet activation was assessed via thrombospondin-1 (TSP-1), neutrophil activating peptide-2 (NAP-2), and platelet factor-4 (PF-4). Although we also attempted to measure platelet-derived growth factor AB (PDGF-AB) and PDGF-BB, these markers were excluded because of undetectable baseline levels in 16 % (N = 12) and 27 % (N = 20) patients, respectively. In addition, brain-derived neurotrophic factor (BDNF) was quantified. All assays were conducted on high-sensitivity enzyme-linked immunosorbent assay (ELISA) platforms from Merck Millipore (Luminex), Bio-Techne SA (Chatillon-sur-Seiche, France), and Tecan France (Lyon, France). The inter-plate coefficient of variation (CV) was calculated to ensure data quality, with all assays exhibiting low variability (CV < 5 %).

### 2.5 Statistical analysis

All statistical analyses were performed using R version 4.3.3 (R foundation for Statistical Computing, Vienna, Austria). Initial data checks included assessing normality of continuous variables using the Kolmogorov-Smirnoff test. Variables that deviated from normality were log-transformed to approximate a Gaussian distribution. Given the repeated-measures design, outliers were identified using Mahalanobis distance and removed to reduce the influence of extreme values. Baseline difference between groups (SI vs no SI and SA vs no SA) were explored using independent samples t-tests or Welch's *t*-test for continuous variables, and χ^2^ tests for categorical variables.

To examine changes in inflammatory, platelet activation, and neurotrophic markers over time, growth curve models were constructed with these markers (TNF-α, MIP-β, TSP-1, NAP-2, PF-4, BDNF, and sIL-2Rα) as dependent variables. Time points (−10, +30, +60, +90, and +120 min) and SI or SA status, along with their interactions (time∗SI or time∗SA), were included as fixed effects, while random intercepts and slopes were specified at the participant level to account for individual variability over time. Then, separate models were conducted for SI and SA, each adjusted across three covariate-adjusted models to account for potential confounders: Model 1 (baseline) included age and BMI; Model 2 added CTQ total scores; and Model 3 further adjusted for IDS-C total scores. Then, in models adjusted for age, BMI, CTQ score, and IDS-C score, polynomial terms for time (up to cubic) and interaction effects were included if they met a significance threshold of p < 0.10, allowing to capture non-linear trends. For significant interaction effects, simple effects for polynomial terms were further examined within each group. Pairwise comparisons, adjusted using the False Discovery Rate (FDR) correction, specified where the differences occurred across time points.

To quantify cumulative responses over time, the Area Under the Curve with respect to ground (AUCg) was calculated for each inflammatory, platelet activation and neurotrophic marker (Pruessner et al., 2003). Logistic regression models were then used to examine association between AUCg values and SI or SA, adjusted for age, BMI, childhood trauma, and depression severity.

A significance level of p < 0.05 was set for all analyses, with FDR correction applied to multiple comparisons in pairwise analyses.

## 3 Results

### 3.1 Sample characteristics

In comparison to participants without SI, those with recent (within past 8 days) SI reported fewer years of education (t_74_ = 2.22, p = 0.030) and higher depression severity, as indicated by the IDS-C scores (t_74_ = −2.54, p = 0.013). Participants with SA history also reported fewer years of education (t_74_ = 3.18, p = 0.002) and had higher CTQ scores (t_72.9_ = −2.56, p = 0.013), compared with participants without SA history. A significant overlap was noted between recent SI and SA groups, with 31 participants meeting criteria for both (χ^2^ = 22.3, p < 0.001). No significant differences were observed in other psychopathology, medication use, gynaecological history, or other sociodemographic variables between groups. At baseline, we observed lower BDNF levels in people with SA history compared to those without, while individuals with SI had higher TNF- α levels compared to people without SI (Table 1).

**Table 1:** Sample description.

	Suicidal ideation	No suicidal ideation	*p*-value	Suicide attempt history	No suicide attempt history	*p-*value
Number of participants	36	40		44	32	

***Sociodemographic***						
Age, years	37.06 ± 2.12	42.03 ± 1.72	0.074	37.18 ± 1.94	43.04 ± 1.93	0.029
Education, years	13.08 ± 0.38	14.35 ± 0.42	0.030	13.00 ± 0.36	14.83 ± 0.42	0.002
Body mass index	24.02 ± 0.62	26.19 ± 1.18	0.167	24.02 ± 0.62	26.19 ± 1.18	0.143
Not in a relationship, n (%)	5 (13.9 %)	8 (20.0 %)	0.480	9 (20.5 %)	4 (12.5 %)	0.363
Has children, n (%)	21 (58.3 %)	23 (57.5 %)	0.941	24 (54.5 %)	20 (62.5 %)	0.488
Not working, n (%)	16 (44.4 %)	24 (60.0 %)	0.175	22 (50.0 %)	18 (56.3 %)	0.590
Current smoker, n (%)	5 (13.9 %)	4 (10.0 %)	0.794	4 (9.1 %)	5 (15.6 %)	0.240

***Medication***						
Antidepressants, n (%)	24 (66.7 %)	32 (80.0 %)	0.188	31 (70.5 %)	25 (78.1 %)	0.453
Benzodiazepine, n (%)	26 (72.2 %)	23 (57.5 %)	0.181	30 (68.2 %)	19 (59.4 %)	0.428
Antiepileptic, n (%)	6 (37.5 %)	10 (25.0 %)	0.374	7 (43.8 %)	9 (28.1 %)	0.197
Antipsychotics, n (%)	13 (36.1 %)	17 (42.5 %)	0.569	19 (43.2 %)	11 (34.4 %)	0.438
Lithium, n (%)	4 (11.1 %)	8 (20.0 %)	0.289	4 (9.1 %)	8 (25.0 %)	0.060
Medication load	3.61 ± 0.37	4.03 ± 0.28	0.369	3.77 ± 0.67	3.93 ± 0.34	0.775

***Psychiatric assessment***						
Bipolar disorder, n (%)	10 (28.6 %)	15 (37.5 %)	0.413	13 (30.2 %)	12 (37.5 %)	0.509
Current anxiety, n (%)	25 (73.5 %)	26 (65.0 %)	0.429	31 (72.1 %)	20 (64.5 %)	0.487
Psychiatric hospitalisations	2.17 ± 0.56	3.23 ± 0.76	0.276	3.23 ± 0.67	1.97 ± 0.72	0.223
IDS-C score	31.08 ± 2.33	22.68 ± 2.34	0.013	27.50 ± 2.07	26.53 ± 3.01	0.568
CTQ total score	54.26 ± 3.34	46.88 ± 2.47	0.080	54.53 ± 2.97	44.57 ± 2.44	0.013
Suicidal ideation, n (%)	–	–	–	31 (70.5 %)	5 (15.6 %)	0.001
Suicide attempt history, n (%)	31 (86.1 %)	13 (32.5 %)	0.001	–	–	–

***Gynecologic history***						
Menopause, n (%)	7 (19.4 %)	9 (22.5 %)	0.744	9 (20.5 %)	7 (21.9 %)	0.881
Irregular menses, n (%)	4 (16.7 %)	8 (34.8 %)	0.154	6 (21.4 %)	6 (21.4 %)	0.434
Contraceptives, n (%)	17 (58.6 %)	13 (46.4 %)	0.357	18 (51.4 %)	12 (51.4 %)	0.819

***Baseline biomarker levels***						
MIP-β, pg/ml	25.80 ± 21.36	26.06 ± 33.37	0.485	22.60 ± 14.74	30.75 ± 39.67	0.122
BDNF, pg/ml	760.39 ± 704.06	887.47 ± 939.00	0.257	662.99 ± 535.63	1064.20 ± 1099.59	0.020
TNF-α, pg/ml	6.75 ± 5.11	4.85 ± 2.70	0.037	6.38 ± 5.08	5.02 ± 2.42	0.103
TSP-1, ng/ml	101.85 ± 69.64	107.72 ± 60.20	0.349	100.47 ± 70.31	111.31 ± 55.65	0.241
NAP-2, ng/ml	183.75 ± 111.26	182.13 ± 110.50	0.950	177.99 ± 108.76	191.62 ± 113.35	0.289
PF-4, ng/ml	81.87 ± 33.74	86.68 ± 33.96	0.272	81.87 ± 33.96	88.43 ± 34.76	0.196
sIL-2Rα, pg/ml	328.79 ± 197.72	359.02 ± 139.15	0.249	350.54 ± 145.15	334.68 ± 201.03	0.362

Abbreviations: IDS-C, Inventory of Depression Symptomatology, clinician-rated; CTQ, Childhood Trauma Questionnaire. MIP-β, macrophage inflammatory protein beta; TSP-1, thrombospondin 1; NAP-2, neutrophil-activating peptide 2; PF-4, platelet factor 4; BDNF, brain-derived neurotrophic factor; TNF-α, tumor necrosis factor alpha; sIL-2Rα, soluble interleukin 2 receptor α.

### 3.2 Temporal dynamics of inflammatory, neurotrophic and platelet activation markers during social stress in individuals with and without suicidal ideation

A significant time effect for sIL-2Rα emerged in models including the SI factor, with levels increasing from baseline to +30 min post-stressor, decreasing between +30 and + 90 min, and rising again at +120 min (F_4,227_ = 2.98, p = 0.020, cubic term: B(SE) = 0.02 (0.01), p = 0.024; Supplementary Fig. 1). No significant time effects were observed for other markers across the full sample.

In models adjusted for age, BMI, childhood trauma, and depression severity, TNF-α levels were significantly higher in participants with recent SI than in those without (0.82 ± 0.05 vs. 0.61 ± 0.05, F_1,53_ = 4.46, p = 0.039). AUC analysis also showed that participants with higher TNF-α AUCg values were significantly more likely to report recent SI (OR = 2.32, 95 % CI [1.02–5.27], p = 0.045). Other markers did not show significant overall differences between SI and non-SI groups (see Supplemental Table 1 for detailed mixed model results).

Examining time∗SI interactions, significant effects emerged for TNF-α (F_4,228_ = 5.92, p < 0.001) and TSP-1 (F_4,274_ = 2.58, p = 0.038) across adjusted models. NAP-2 lost significance as covariates were added. Polynomial analysis (Supplemental Table 3) indicated differences in temporal dynamics in participants with vs without recent SI, showing distinct trajectories for TNF-α (cubic term, t_228_ = 4.76, p < 0.001), BDNF (linear term, t_275_ = 2.39, p = 0.017), MIP-1β (cubic term, t_235_ = 2.12, p = 0.035), TSP-1 (quadratic term, t_274_ = −2.42, p = 0.016), NAP-2 (quadratic term, t(_274_) = −2.43, p = 0.016), and PF-4 (quadratic term, t_274_ = −2.25, p = 0.025).

Specifically, in participants without, but not with, recent SI, TNF-α and MIP-1β followed cubic patterns, showing a reduction from baseline to +30 min, an increase from +30 to +90 min, and a decrease from +90 to +120 min (TNF-α: B (SE) = −0.13 (0.03), p < 0.001; MIP-β: B (SE) = −0.03 (0.02), p = 0.048; Fig. 1A and C). For TNF-α, pairwise comparisons within participants without recent SI confirmed significant temporal changes across these intervals, with a significant decrease from −10min to +30min (p = 0.004), an increase from +30min to +90min (p = 0.001), and a decrease again from +90min to +120min (p = 0.009). MIP-1β comparisons showed no significant within-group differences. In contrast, BDNF in participants with recent SI showed a significant positive linear increase over time (B (SE) = 0.12 (0.04), p = 0.009), with significant pairwise changes from −10 min to +90 min in SI (p = 0.043), while there were no significant shifts in participants without recent SI (Fig. 1B).

**Fig. 1:**
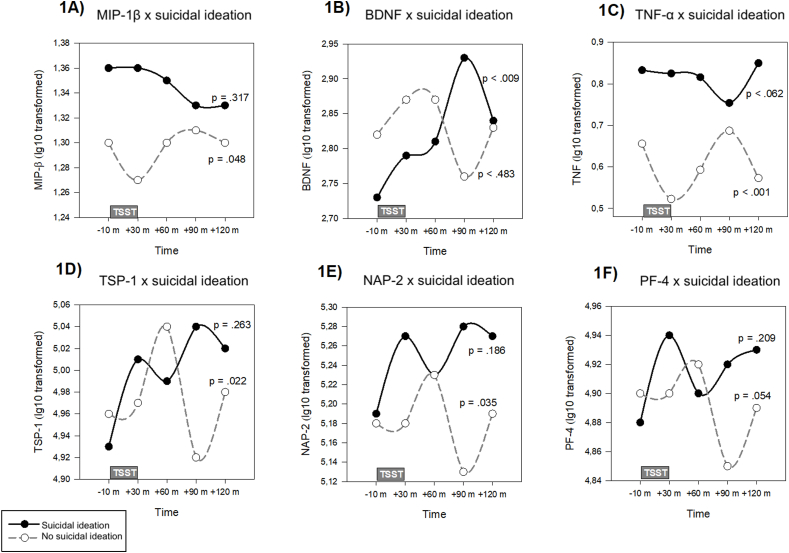
Temporal patterns of inflammatory, platelet activation, and neurotrophic markers during social stress in participants with and without recent suicidal ideation.

Platelet activation markers TSP-1 and NAP-2 showed a quadratic trajectory only in participants without recent SI, characterized by no immediate response at +30 min, followed by an increase from +30 to +60 min, a decline from +60 to +90 min, and a subsequent increase at +120 min (TSP-1: B (SE) = 0.08 (0.03), p = 0.022; NAP-2: B (SE) = 0.07 (0.03), p = 0.001; Fig. 1D and E). In pairwise comparisons, TSP-1 significantly decreased from +60 to +90 min in participants without recent SI (p = 0.021). No significant time∗SI interactions were identified for other inflammatory, platelet activation, or neurotrophic markers (Supplemental Table 1).

### 3.3 Temporal dynamics of inflammatory, neurotrophic and platelet activation markers during social stress in individuals with and without suicide attempt history

In the analysis on SA history, growth curve models showed a significant effect of time on sIL-2Rα, with levels following a cubic trajectory across the TSST (F_4,227_ = 3.01, p = 0.019, B (SE) = 0.02 (0.01), p = 0.021) when including SA factor. However, the main SA effect was not significant for any of the markers. There were also no biomarkers associated with SA in AUC analysis.

**Fig. 2:**
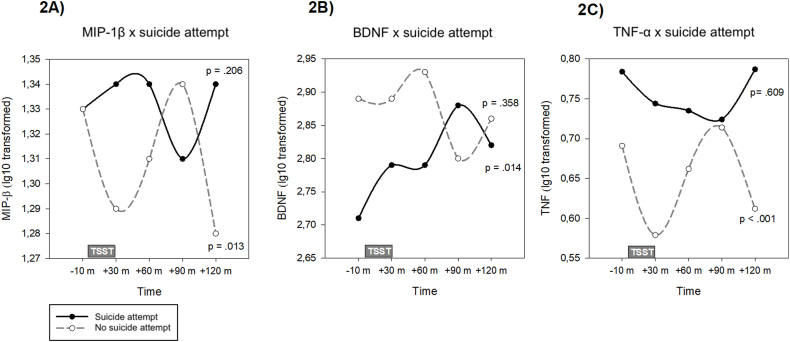
Temporal patterns of inflammatory, platelet activation, and neurotrophic markers during social stress in participants with and without suicide attempt history.

We found no significant time∗SA interactions in global mixed models. However, polynomial analyses indicated distinct temporal patterns, showing a linear effect for BDNF (t_275_ = 2.28, p = 0.024) and cubic effects for MIP-β (t_235_ = 2.74, p = 0.007) and TNF-α (t_228_ = 3.15, p = 0.030) (Supplemental Table 2). For BDNF, participants with SA history, but not those without it, exhibited a significant positive linear increase over time (B(SE) = 0.09 (0.04), p = 0.014) (Fig. 2B). Meanwhile, MIP-β and TNF-α followed cubic trajectories in participants without SA history only, showing a decline from baseline to +30 min, followed by an increase to +90 min, and a subsequent decrease to +120 min (MIP-β: B (SE) = −0.05 (0.02), p = 0.013β; TNF-α: B (SE) = −0.11(0.03), p < 0.001 for TNF-α) (Fig. 2A and C). Among suicide attempters, TNF-α showed a quadratic response, with levels decreasing from baseline to +90 min and rising again at +120 min (B (SE) = 0.05 (0.03), p = 0.038). Pairwise comparisons for TNF-α in participants without SA history showed decreases from baseline to +30 min (p = 0.050) and increases from +30 to +90 min (p = 0.050). No other markers displayed significant effects or trends for the time∗SA interaction.

## 4 Discussion

The ability to terminate an acute stress response effectively is highly adaptive, reducing vulnerability to stress-related disease (de Kloet et al., 2005). In this study, we observed distinct patterns of inflammatory, platelet, and neurotrophic markers in response to an acute social stress test in women with depression and SA history or recent SI versus those without. We found overall elevated TNF-α levels during the social stress in participants with recent SI, alongside a cubic trajectory in pro-inflammatory markers TNF-α and MIP-1 β in participants without recent SI or SA, but not in those with it. Conversely, a linear increase in the neurotrophic factor BDNF was observed in individuals with recent SI or SA. Participants with recent SI also showed an absence of the quadratic response in platelet activation markers (TSP-1, PF-4, and NAP-2) seen in patients without recent SI. These findings suggest that STB may be characterized not only by baseline biological differences but also by an impaired ability to dynamically modulate inflammatory, platelet, and neurotrophic responses in acute social stress.

### 4.1 Dynamic responses of pro-inflammatory cytokines and chemokines

Overall increased levels of TNF-α emerged as a key differentiator between participants with SA history or recent SI and those without. TNF-α, a pro-inflammatory cytokine, increases during acute stress and has been linked to increased blood-brain barrier permeability and neuroinflammatory processes (Cheng et al., 2018; Hochman et al., 2023). These results align with prior studies implicating peripheral inflammation in STB, indicating that elevated pro-inflammatory signalling may contribute to the emotional dysregulation and cognitive disturbances commonly observed in individuals with STB (Colmenero-Navarrete et al., 2022; Lengvenyte et al., 2019; Miller et al., 2023; Moriarity et al., 2023). An increase in a soluble receptor for TNF-α during a social stress task has been associated with greater activity in the dorsal anterior cingulate cortex and anterior insula, brain regions that have been associated with processing rejection-related distress and negative affect (Slavich et al., 2010).

Interestingly, participants without SI or SA, but not those with it, exhibited a cubic response pattern in TNF-α, and, to a lesser degree, in another pro-inflammatory marker MIP-1 β, characterized by an initial reduction, inflammatory rebound, and then recovery. The biphasic TNF-α response, noted previously in healthy and depressed individuals, may indicate a regulated inflammatory response to acute stress (Marsland et al., 2017). The lack of this pattern in individuals with STB could suggest a diminished capacity for dynamic immune regulation under stress. Indeed, a previous meta-analysis has reported that healthy controls showed higher IL-6 and, to a lower extent, TNF-α, increase in response to social stress (Marsland et al., 2017). While, due to a large portion of undetectable values, we could not perform statistical analyses on IL-6, we found overall higher TNF-α levels in suicide attempters and ideators, which almost did not change, while the response was variable in the control group.

This may reflect glucocorticoid resistance, a state where, due to frequent or chronic social-environmental threat-related stress, immune cells become less responsive to cortisol's anti-inflammatory effects, thus impairing coordination between the HPA axis and pro-inflammatory cytokine activity (McInnis et al., 2015; Slavich and Irwin, 2014). **Allostatic load, reflecting the biological impact of chronic stress, has also been associated with suicide** (Gou et al., 2024).

### 4.2 Differences in brain-derived growth factor response

Interestingly, we found a linear increase in BDNF levels during acute social stress only in individuals with a history of SA and recent SI. BDNF, a neurotrophic factor critical for neuronal plasticity and resilience, has counteracting effects of excessive stress-induced glucocorticoid signalling (Duman et al., 2016). While it is traditionally viewed as protective, elevated BDNF in response to stress could reflect a maladaptive attempt to restore neural homeostasis. Previous research indicates that BDNF response to stress is closely coupled with cortisol regulation, enabling a balanced response to stress, and desynchronization of BDNF-TrkB and glucocorticoid-receptor signalling pathways contribute to stress maladaptation (Gray et al., 2013; Jeanneteau et al., 2019). Interestingly, a previous study associated higher cortisol stress reactivity and steeper BDNF recovery after stress (Linz et al., 2019). Meanwhile, individuals with STB tend to have blunted cortisol response (O'Connor et al., 2017; Shalev et al., 2019). Therefore, the increase in BDNF levels in people with STB during social stress may represent a compensatory but dysregulated neuroplastic response to acute stress, possibly reflecting broader neuroplasticity deficits in the context of STB.

### 4.3 Differences in dynamical responses of platelet activation and vascular homeostasis markers

We also found differences in dynamical responses to social stress of platelet-related markers, which showed quadratic response patterns in individuals without SI but not in those with SI. TSP-1, PF-4, and NAP-2 are stored and released from platelets, which play a critical role in later phases of the stress response, involving hemostasis, immune modulation, and tissue repair (Cognasse et al., 2022; Koupenova et al., 2018). Mental stress is linked to increased platelet activity (Koudouovoh-Tripp et al., 2021). Platelets may also act as a relay between brain and periphery, and are implicated in central nervous system inflammation (Leiter and Walker, 2020). The observed quadratic response only in individuals without SI may suggest a healthy regulatory system, where platelet activation is modulated to prevent excessive aggregation and support vascular stability (Gupta et al., 2020). In contrast, the absence of these dynamical fluctuations in individuals with SI could indicate systemic rigidity in response to stress.

TSP-1, in particular, is a stress-responsive glycoprotein, serving as the primary activator of TGF-β, and affecting cell adhesion, cell migration, growth factor, angiogenesis, nitric oxide signalling and responses of cells to stress (Kaur and Roberts, 2024). It also plays a role in neuron-astrocyte communication, and TSP-1 effects in medial prefrontal cortex have been shown to mediate stress resilience in mice (Gao et al., 2023; Nagai et al., 2019). A recent study has linked increased plasma levels of TSP-1 with suicidal events during a two-year follow-up, but not SI at intake or SA history, further underscoring the dynamical connection between it and STB (Lengvenyte et al., 2023). Although we did not perform statistical analyses on PDGF-AB due to a substantial portion of undetectable values, this previous study demonstrated a strong correlation between TSP-1 and PDGF-AB (Lengvenyte et al., 2023). Thus, TSP-1 may serve as a surrogate indicator for PDGF-related pathways in the context of STB. However, future studies are warranted to reliably quantify PDGF-AB and PDGF-BB and further elucidate their changes in response to stress in context of STB.

Meanwhile, while we previously found no cross-sectional evidence in STB, depression has been associated with higher blood levels of NAP-2 and PF-4 (Leighton et al., 2018; Lengvenyte et al., 2023; Musselman et al., 2000). PF-4 administration attenuated age-related hippocampal neuroinflammation in mice, suggesting a possibly causal association with brain health (Schroer et al., 2023).

### 4.4 Dynamical biological system dysregulation

Suicidal thoughts are characterized by rapid onset, high variability and dynamic fluctuations, aligning with theories conceptualizing suicide as an escape from aversive internal states (Wang et al., 2023). Complex dynamical systems are composed of many components and characterized by nonlinear interactions, feedback loops, and dynamical change over time (Olthof et al., 2023; Wang et al., 2023). Since brain and peripheral inflammatory responses are clearly tightly co-regulated, these components are conceivably both psychological and biological (Jin et al., 2024). Therefore, STB may be understood within the framework of dynamical systems theory, with adaptive biomarker fluctuations in response to stress indicating resilience and capacity to respond, regulate, and recover from social stress in participants without SI or SA. In contrast, the rigidity of these responses in participants with STB could indicate systemic dysregulation of homeostatic mechanisms, potentially driven by chronic stress and low-grade inflammation, that often reported in STB (Gou et al., 2024; Lengvenyte et al., 2023; Neupane et al., 2023). In line with the reduction of adaptive capacity, suicide attempters have been shown to have decreased high frequency heart rate variability in response to social stress, further suggesting the reduced dynamical physiological system adaptation capacity during social stress situations (Wilson et al., 2016).

## 5 Limitations

This study has several limitations. First, the cross-sectional nature of the study prevents us from drawing conclusions about the causality and directionality of observed differences, and limits our ability to see whether observed perturbations persist after the acute stress. Second, the moderate sample size may limit the generalizability of the findings. To reduce heterogeneity, we only included females with depression, which also underscores the generalizability. Replication in larger and more diverse populations is essential. Future studies should include both sexes, more diverse psychiatric conditions, as well as individuals without psychiatric disorders. Given that our sample was moderate in size, we decided to exclude markers for which a substantial proportion of samples yielded undetectable values (IL-6, PDGF-AB, and PDGF-BB), as including them could have compromised the robustness of our analyses. Future studies with larger samples and optimized assay sensitivity are needed to reliably measure these markers and further elucidate their role in STB. In addition, while we performed analyses for recent SI and SA, there was a significant overlap between the two groups, but sample size did not allow us to perform further more granular analyses and identify responses specific SI without SA history and vice versa. Medication use is another potential confounding factor, as antidepressants and other medications could influence stress response of studied markers. While we did not see differences based on medication groups or medication load, more fine-grained analyses are needed, and future research should explore how specific medications and dosages may affect the inflammatory and neurotrophic responses in suicidal individuals. Furthermore, while we did not observe differences in SI and SA according to hormonal contraception, previous research has showed stress response differences in females under hormonal contraception, and the implication of sex hormones in the observed differences needs further study (Mengelkoch et al., 2024).

## 6 Conclusions

Our findings suggest that STB may be associated with altered inflammatory, platelet, and neurotrophic responses to acute stress in women with depression, marked by higher overall TNF-α levels in participants with recent SI, absence of cubic responses in inflammatory markers, a linear BDNF increase in those with recent SI or SA history, and a lack of quadratic response in platelet markers in participants with recent SI. This highlights the importance of dynamic biomarker assessments and a possibly impaired dynamical adaptation of biological systems in individuals with STB. Suicidal crises are often acute, and static measures of biological dysfunction may not fully capture the transient yet critical shifts that occur during stress. Future longitudinal studies should explore real-time biomarker monitoring in response to acute stressors to capture dynamic changes linked to STB. Future studies should also integrate stress response markers with inflammatory, platelet activation, and neurotrophic markers to gain deeper insights into stress system dysregulation in individuals with STB. Interventions combining psychological stress reduction and pharmacological modulation of immune and neurotrophic pathways may provide insight into causal mechanisms and potential reversibility of these dysregulations.

## CRediT authorship contribution statement

**Aiste Lengvenyte:** Conceptualization, Formal analysis, Methodology, Visualization, Writing – original draft. **Emilie Olié:** Conceptualization, Investigation, Writing – review & editing. **Fabrice Cognasse:** Funding acquisition, Investigation, Methodology, Writing – review & editing. **Hind Hamzeh-Cognassse:** Investigation, Methodology. **Adrian Alacreu-Crespo:** Formal analysis, Methodology, Supervision, Visualization, Writing – original draft, Writing – review & editing. **Philippe Courtet:** Conceptualization, Funding acquisition, Resources, Supervision, Writing – review & editing.

## Declaration of competing interest

All authors report no actual or potential conflict of interest that could influence or bias their work.

## Figures and Tables

**figs1:**
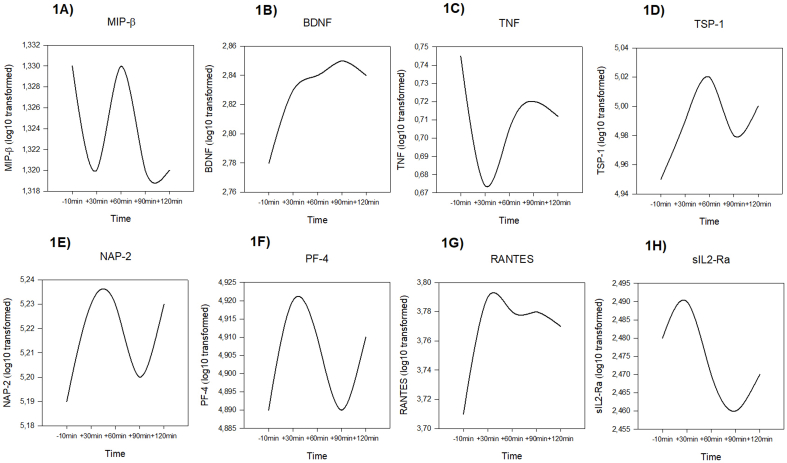


## Data Availability

The data that has been used is confidential.
