# Supplementary material for: Temporal dynamics of inflammatory, platelet, and neurotrophic markers during social stress in relation to suicidal ideation and suicide attempt history
Source: Brain Behav Immun Health. 2025 Mar 24;45:100984. doi: 10.1016/j.bbih.2025.100984 (PMC11985145; doi:10.1016/j.bbih.2025.100984)
Supplement: Multimedia component 1 [file mmc1.docx]

**Supplemental Table 1. Mixed-Effects Models Examining Temporal Changes in Inflammatory, Platelet Activation, and Neurotrophic Markers in Relation to Recent Suicidal Ideation**

|  | Model 1 | Model 2 | Model 3 |
| --- | --- | --- | --- |
| MIP-β | SI: F_1,64_= 1.73, p = 0.194  Time: F_4,240_= 0.48, p = 0.754  **SI*Time: F_4,240_= 2.35, p = 0.055** | SI: F_1,64_= 1.64, p = 0.205  Time: F_4,236_= 0.50, p = 0.736  **SI*Time: F_4,236_= 2.19, p = 0.071** | SI: F_1,63_= 1.36, p = 0.249  Time: F_4,236_= 0.44, p = 0.775  **SI*Time: F_4,236_= 2.20, p = 0.069** |
| BDNF | SI: F_1,69_= 0.42, p = 0.517  Time: F_4,274_= 1.01, p = 0.405  **SI*Time: F_4,278_= 2.03, p = 0.089** | SI: F_1,67_= 0.62, p = 0.511  Time: F_4,274_= 0.82, p = 0.511  **SI*Time: F_4,274_= 2.27, p = 0.062** | SI: F_1,66_= 0.65, p = 0.425  Time: F_4,274_= 0.82, p = 0.513  **SI*Time: F_4,274_= 2.27, p = 0.062** |
| TNF-$\boldsymbol{\alpha}$ | **SI: F_1,56_= 5.46, p = 0.023**  Time: F_4,232_= 1.76, p = 0.137  **SI*Time: F_4,228_= 6.02, p < .001** | **SI: F_1,54_= 4.51, p = 0.038**  Time: F_4,228_= 1.86, p = 0.118  **SI*Time: F_4,228_= 5.94, p < .001** | **SI: F_1,53_= 4.46, p = 0.039**  Time: F_4,228_= 1.86, p = 0.119  **SI*Time: F_4,228_= 5.92, p < .001** |
| TSP-1 | SI: F_1,69_= 1.39, p = 0.242  Time: F_4,278_= 1.65, p = 0.161  **SI*Time: F_4,278_= 2.06, p = 0.086** | SI: F_1,67_= 1.01, p = 0.319  Time: F_4,274_= 1.39, p = 0.236  **SI*Time: F_4,274_= 2.58, p = 0.037** | SI: F_1,66_= 0.95, p = 0.334  Time: F_4,274_= 1.39, p = 0.237  **SI*Time: F_4,274_= 2.58, p = 0.038** |
| NAP-2 | SI: F_1,69_= 1.24, p = 0.269  Time: F_4,274_= 0.98, p = 0.419  **SI*Time: F_4,278_= 2.45, p = 0.047** | SI: F_1,67_= 0.92, p = 0.341  Time: F_4,274_= 0.77, p = 0.547  **SI*Time: F_4,274_= 2.40, p = 0.051** | SI: F_1,66_= 0.90, p = 0.345  Time: F_4,274_= 0.77, p = 0.548  **SI*Time: F_4,274_= 2.39, p = 0.051** |
| PF-4 | SI: F_1,69_= 0.01, p = 0.912  Time: F_4,274_= 1.13, p = 0.341  **SI*Time: F_4,278_= 2.15, p = 0.074** | SI: F_1,67_= 0.01, p = 0.999  Time: F_4,274_= 0.98, p = 0.417  **SI*Time: F_4,274_= 2.20, p = 0.069** | SI: F_1,66_= 0.01, p = 0.997  Time: F_4,274_= 0.98, p = 0.419  **SI*Time: F_4,274_= 2.19, p = 0.070** |
| RANTES | SI: F_1,69_= 0.01, p = 0.993  Time: F_4,274_= 0.77, p = 0.541  SI*Time: F_4,278_= 1.70, p = 0.149 | SI: F_1,67_= 0.01, p = 0.956  Time: F_4,274_= 0.68, p = 0.608  SI*Time: F_4,274_= 1.83, p = 0.122 | SI: F_1,66_= 0.01, p = 0.609  Time: F_4,274_= 0.68, p = 0.609  SI*Time: F_4,274_= 1.83, p = 0.123 |
| sIL-2Rα | **SI: F_1,56_= 4.15, p = 0.046**  **Time: F_4,231_= 3.01, p = 0.019**  SI*Time: F_4,231_= 0.25, p = 0.907 | **SI: F_1,54_= 3.22, p = 0.074**  **Time: F_4,227_= 2.98, p = 0.019**  SI*Time: F_4,227_= 0.27, p = 0.898 | **SI: F_1,53_= 3.34, p = 0.073**  **Time: F_4,227_= 2.98, p = 0.020**  SI*Time: F_4,227_= 0.27, p = 0.898 |

Abbreviations: MIP-β, macrophage inflammatory protein beta; TSP-1, thrombospondin 1; NAP-2, neutrophil-activating peptide 2; RANTES, regulated upon activation normal T cell expressed and presumably secreted; PF-4, platelet factor 4; BDNF, brain-derived neurotrophic factor; TNF-$\alpha$, tumor necrosis factor alpha; sIL-2Rα, soluble interleukin 2 receptor α.

**Model 1:** adjusted for age and body mass index

**Model 2:** adjusted for age, body mass index, and Childhood Trauma Questionnaire score

**Model 3:** adjusted for age, body mass index, Childhood Trauma Questionnaire score, and IDS-C score
