# Supplementary material for: Temporal dynamics of inflammatory, platelet, and neurotrophic markers during social stress in relation to suicidal ideation and suicide attempt history
Source: Brain Behav Immun Health. 2025 Mar 24;45:100984. doi: 10.1016/j.bbih.2025.100984 (PMC11985145; doi:10.1016/j.bbih.2025.100984)
Supplement: Multimedia component 2 [file mmc2.docx]

**Supplemental Table 2. Mixed-Effects Models Examining Temporal Changes in Inflammatory, Platelet Activation, and Neurotrophic Markers in Relation to Suicide Attempt History**

|  | Model 1 | Model 2 | Model 3 |
| --- | --- | --- | --- |
| MIP-β | SA: F_1,64_= 0.14, p = 0.710  Time: F_4,240_= 0.45, p = 0.771  SA*Time: F_4,240_= 2.27, p = 0.063 | SA: F_1,64_= 0.09, p = 0.759  Time: F_4,236_= 0.48, p = 0.749  SA*Time: F_4,236_= 2.21, p = 0.069 | SA: F_1,63_= 0.02, p = 0.897  Time: F_4,236_= 0.43, p = 0.789  SA*Time: F_4,236_= 2.26, p = 0.064 |
| BDNF | SA: F_1,69_= 2.77, p = 0.100  Time: F_4,274_= 1.02, p = 0.396  SA*Time: F_4,278_= 1.94, p = 0.104 | SA: F_1,67_= 3.23, p = 0.077  Time: F_4,274_= 0.84, p = 0.501  SA*Time: F_4,274_= 2.09, p = 0.082 | SA: F_1,66_= 3.22, p = 0.077  Time: F_4,274_= 0.84, p = 0.502  SA*Time: F_4,274_= 2.09, p = 0.083 |
| TNF-$\boldsymbol{\alpha}$ | SA: F_1,56_= 0.13, p = 0.716  Time: F_4,232_= 1.34, p = 0.182  SA*Time: F_4,228_= 2.33, p = 0.057 | SA: F_1,54_= 0.01, p = 0.961  Time: F_4,228_= 1.67, p = 0.156  SA*Time: F_4,228_= 2.37, p = 0.053 | SA: F_1,53_= 0.01, p = 0.994  Time: F_4,228_= 1.67, p = 0.157  SA*Time: F_4,228_= 2.36, p = 0.054 |
| TSP-1 | SA: F_1,69_= 0.02, p = 0.900  Time: F_4,278_= 1.66, p = 0.159  SA*Time: F_4,278_= 1.10, p = 0.357 | SA: F_1,67_= 0.01, p = 0.991  Time: F_4,274_= 1.39, p = 0.238  SA*Time: F_4,274_= 1.13, p = 0.342 | SA: F_1,66_= 0.01, p = 0.998  Time: F_4,274_= 1.39, p = 0.239  SA*Time: F_4,274_= 1.13, p = 0.344 |
| NAP-2 | SA: F_1,69_= 0.09, p = 0.770  Time: F_4,274_= 1.03, p = 0.392  SA*Time: F_4,278_= 1.29, p = 0.272 | SA: F_1,67_= 0.06, p = 0.815  Time: F_4,274_= 0.81, p = 0.522  SA*Time: F_4,274_= 1.14, p = 0.338 | SA: F_1,66_= 0.05, p = 0.819  SA*Time: F_4,274_= 1.14, p = 0.339  Time: F_4,274_= 0.80, p = 0.524 |
| PF-4 | SA: F_1,69_= 0.08, p = 0.779  Time: F_4,274_= 1.15, p = 0.333  SA*Time: F_4,278_= 0.78, p = 0.538 | SA: F_1,67_= 0.13, p = 0.724  Time: F_4,274_= 0.99, p = 0.411  SA*Time: F_4,274_= 0.75, p = 0.562 | SA: F_1,66_= 0.12, p = 0.723  Time: F_4,274_= 0.99, p = 0.412  SA*Time: F_4,274_= 0.74, p = 0.563 |
| RANTES | SA: F_1,69_= 2.67, p = 0.107  Time: F_4,274_= 0.77, p = 0.543  SA*Time: F_4,278_= 1.26, p = 0.285 | SA: F_1,67_= 2.65, p = 0.108  Time: F_4,274_= 0.67, p = 0.614  SA*Time: F_4,274_= 1.19, p = 0.314 | SA: F_1,66_= 2.72, p = 0.104  Time: F_4,274_= 0.67, p = 0.615  SA*Time: F_4,274_= 1.18, p = 0.320 |
| sIL-2Rα | SA: F_1,56_= 1.09, p = 0.299  **Time: F_4,231_= 3.06, p = 0.017**  SA*Time: F_4,231_= 1.29, p = 0.274 | SA: F_1,54_= 0.69, p = 0.410  **Time: F_4,227_= 3.02, p = 0.019**  SA*Time: F_4,227_= 1.32, p = 0.265 | SA: F_1,53_= 0.62, p = 0.435  **Time: F_4,227_= 3.01, p = 0.019**  SA*Time: F_4,227_= 1.31, p = 0.266 |

Abbreviations: MIP-β, macrophage inflammatory protein beta; TSP-1, thrombospondin 1; NAP-2, neutrophil-activating peptide 2; RANTES, regulated upon activation normal T cell expressed and presumably secreted; PF-4, platelet factor 4; BDNF, brain-derived neurotrophic factor; TNF-$\alpha$, tumour necrosis factor alpha; sIL-2Rα, soluble interleukin 2 receptor α.

**Model 1:** adjusted for age and body mass index

**Model 2:** adjusted for age, body mass index, and Childhood Trauma Questionnaire score

**Model 3:** adjusted for age, body mass index, Childhood Trauma Questionnaire score, and IDS-C score.
