# Supplementary material for: Temporal dynamics of inflammatory, platelet, and neurotrophic markers during social stress in relation to suicidal ideation and suicide attempt history
Source: Brain Behav Immun Health. 2025 Mar 24;45:100984. doi: 10.1016/j.bbih.2025.100984 (PMC11985145; doi:10.1016/j.bbih.2025.100984)
Supplement: Multimedia component 3 [file mmc3.docx]

**Supplemental Table 3. Polynomials of significant time and suicidal ideation or suicide attempt interactions**

|  | Linear | Quadratic | Cubic | Quartic |
| --- | --- | --- | --- | --- |
| *Suicidal ideation* |  |  |  |  |
| MIP-β | t_235_= - 0.83, p = 0.406 | t_235_= - 0.62, p = 0.537 | **t_235_= 2.12, p = 0.035** | t_235_= - 0.49, p = 0.618 |
| BDNF | **t_275_= 2.39, p = 0.017** | t_275_= - 0.46, p = 0.649 | **t_275_= - 1.97, p = 0.049** | t_275_= - 1.49, p = 0.135 |
| TNF-$\boldsymbol{\alpha}$ | t_228_= - 0.29, p = 0.770 | t_228_= 0.64, p = 0.521 | **t_228_= 4.76, p < 0.001** | t_228_= 1.01, p = 0.316 |
| TSP-1 | t_274_= 1.31, p = 0.193 | t_274_= - 0.27, p = 0.789 | t_274_= - 0.72, p = 0.472 | **t_274_= - 2.42, p = 0.016** |
| NAP-2 | t_274_= 1.46, p = 0.144 | t_274_= - 0.34, p = 0.732 | t_274_= - 0.23, p = 0.822 | **t_274_= - 2.43, p = 0.016** |
| PF-4 | t_274_= 1.64, p = 0.101 | t_274_= - 0.35, p = 0.729 | t_274_= 0.15, p = 0.884 | **t_274_= - 2.25, p = 0.025** |
| *Suicide attempt history* |  |  |  |  |
| MIP-β | t_235_= 0.38, p = 0.702 | t_235_= 0.57, p = 0.570 | **t_235_= 2.74, p = 0.007** | t_235_= 0.91, p = 0.362 |
| BDNF | **t_275_= 2.28, p = 0.024** | t_275_= - 0.59, p = 0.558 | t_275_= - 1.03, p = 0.303 | t_275_= - 1.88, p = 0.064 |
| TNF-$\boldsymbol{\alpha}$ | t_228_= 0.65, p = 0.948 | t_228_= 1.47, p = 0.144 | **t_228_= 3.15, p = 0.030** | t_228_= 0.03, p = 0.976 |

Abbreviations: MIP-β, macrophage inflammatory protein beta; TSP-1, thrombospondin 1; NAP-2, neutrophil-activating peptide 2; RANTES, regulated upon activation normal T cell expressed and presumably secreted; PF-4, platelet factor 4; BDNF, brain-derived neurotrophic factor; TNF-$\alpha$, tumour necrosis factor alpha; sIL-2Rα, soluble interleukin 2 receptor.
